# Effects of straw mulching combined with nitrogen application on soil organic matter content and atrazine digestion

**DOI:** 10.1038/s41598-022-20097-8

**Published:** 2022-09-23

**Authors:** Wan-feng Zhang, Shu-qing Yang, An Chang, Li-ge Jia, Ji-fang E

**Affiliations:** 1 Inner Mongolia Normal University, Hohhot, 010022 China; 2 Inner Mongolia Agricultural University, Hohhot, 010018 China

**Keywords:** Plant sciences, Environmental sciences

## Abstract

To explore the response of atrazine (AT) degradation rate, soil organic matter (SOM) distribution and the relationship between them to straw mulching and nitrogen application, field experiments were conducted to study the distribution of SOM content and AT degradation rate under different straw returning modes combined with nitrogen fertilization in 2 years in Hetao Irrigation District. No (N_0_), low (N_L_), medium (N_M_), and high (N_H_) levels of N fertilization were incorporated into the soil combined with the surface coverage straw (Treatment BN_0_, BN_L_, BN_M_, BN_H_, respectively) and the deeply buried straw (Treatment SN_0_, SN_L_, SN_M_, SN_H_, respectively). The traditional cultivation was used as a control treatment (Treatment CK). The results showed that SOM content of Treatment B was accumulated in 0~20 cm soil layer. The largest SOM content of Treatments B in 0~20 cm soil layer was found in BN_H_ treatment, with an average increase of 14.2% and 24.1% significantly when compared with those in CK and SN_H_ (*P* < 0.05), respectively. The SOM content of Treatments B increased with the increase of nitrogen application and decreased with the deepening of soil depth. The SOM content of Treatment S was accumulated in the soil layer (20~40 cm and 40~60 cm) near the inter-layer. With the increase of nitrogen application and depth of soil layer, the SOM content increased firstly and then decreased. SN_M_ had the largest SOM content in the soil layer of 20~40 cm and 40~60 cm, with an average increase of 82.6% and 67.7% when compared with Treatment CK (*P* < 0.05). In the soil layer over 60 cm, there was no significant difference in SOM content of different straw returning methods under the same nitrogen level (*P* > 0.05). Straw returning methods and nitrogen application level significantly affected AT digestion rate and digestion half-life, with significant differences among treatments (*P* < 0.05). It found that treatment SN_M_ had the highest digestion rate and the shortest half-life of AT. Compared with Treatment CK, the digestion rate of Treatment SN_M_ was increased by 5.3% on average, and the half-life was shortened by 3.9 days on average. Single regression and stepwise regression analysis of the half-life of AT degradation and SOM content in different soil layers (0~20 cm and 20~40 cm) showed that the degradation of AT was greatly affected by SOM content of 20~40 cm soil layer. Based on the comprehensive analysis, the effect of straw deep burial combined with medium nitrogen application rate (Treatment SN_M_) was best, which could achieve the goal of increasing SOM content and shortening the half-life of AT digestion. The research provided a technical support for straw resource utilization, alleviated AT pollution and improved farmland ecological environment in Hetao Irrigated District.

## Introduction

The scientific name of atrazine is 2-chloro-4-ethylamine-6-isoalanine-1, 3, 5-triazobenzene, also known as atrazine (AT). It is a triazobenzene herbicide widely used at home and abroad^1^, which is suitable for the control of annual grass weeds and broad-leaf weeds, and has a certain inhibitory effect on some perennial weeds^2^. AT is easy to dissolve and has a long residual period. Massive spraying of AT could easily cause soil and water pollution^3^, damage crops^4^, even interfere with human endocrine balance and cause cancer^5^. Studies had shown that the adsorption and digestion of AT in soil were not only related to its own physical and chemical properties, but also affected by water and soil environmental factors such as SOM content, pH value, temperature, microbial quantity, activity, and soil enzymes^6,7^. The rhizosphere effect of plant roots improve the number and activity of soil microorganisms, increases the number of soil culturable bacteria^8^, and direct degradation of AT by secreted and released enzymes^9^.The absorption rate of AT in soil increased with the increase of SOM content^10^. Application of inorganic–organic fertilizer could improve the number and activity of microorganisms in contaminated soil, which was conducive to accelerating biodegradation of organic pollutants^11^, effectively repair contaminated soil, and shorten the digestion half-life of AT in soil^12^.

It had been proved that the tillage application pattern of straw returning and combined application with organic and inorganic fertilizers have good practical effects in improving crop yield and fertilizer utilization efficiency. Studies have shown that straw returning could change soil physical and chemical properties, alleviate soil erosion caused by tillage^13^, alleviate soil degradation caused by excessive fertilization^14^, improve crop yield in dryland and improve soil organic matter content^15^. However, the C/N ratio of corn straw was higher, and straw application alone was likely to cause microbial competition with crops for inorganic nitrogen, and blindly applying a large amount of nitrogen reduces the accumulation of soil carbon, which was not conducive to the accumulation of soil organic matter^16^. Therefore, when straw was returned to field, it was necessary to apply appropriate nitrogen fertilizer to reduce C/N. It could promote inorganic nitrogen accumulation^17^, improve soil available nutrients and microbial content^18^, improve soil organic matter stability^19^, and improve and maintain enzyme activity for a long time^20^.

Compared with previous studies on the effects of straw returning on crop physiological traits and soil straw, there was few reports about the effects of different straw returning modes and nitrogen application coupling on AT digestion and its correlation with SOM spatial distribution. Therefore, the study was a breakthrough point, in that different modes of straw mulching combined with various amounts of N fertilizer applications were explored. The study’s field experiments were carried out in Hetao Irrigation District of Inner Mongolia, China. The experiments examined the effects of straw mulching combined with N fertilizer applications on the SOM spatial distribution, the law of AT digestion, and the correlation between SOM content and the half-life of AT digestion. The results obtained in the research potentially provide some useful references for future improvements in SOM spatial distribution and AT digestion under the different straw mulching combined with reasonable N fertilizer applications, which not only improve the distribution of organic matter in the tillage layer and promote AT digestion, but also alleviate the environmental pollution of AT in farmland and will enrich the current theories regarding returning straw to soil in similar agricultural areas.

## Materials and methods

According to the guidelines for the implementation of the research project and the relevant research rules, all methods were performed in the research.

### Experiment site description

**Figure 1:**
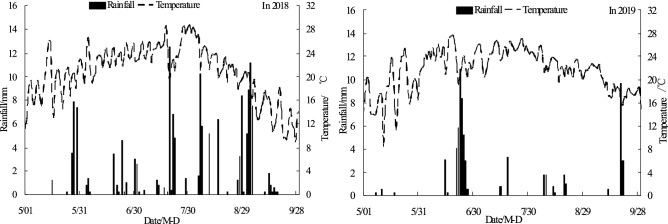
Daily rainfall and temperature during growing period of summer maize.

This experiment plot was located in the Jiuzhuang experimental demonstration area, Hetao Irrigation District, Inner Mongolia, China (40°42′N, 107°24′E, 1040 ~ 1043 m altitude). It is an arid and semi-arid continental climate zone with low annual precipitation of 138 ~ 222 mm, high annual evaporation of 1999 ~ 2346 mm, large temperature changes and the total radiation reaches 6200 MJ·m^−2^. The surface accumulations of salt is serious during spring and winter, which is a typical continental climate zone. The trial was conducted from early May 2018 to late September 2019. In accordance with the triangle map of soil texture (USA), the test soil was determined to be silty loam (the mass ratio of sand, clay and powder was 8:2:15). The basic physical and chemical properties of experimental soil before sowing were shown in Table 1. The observed daily rainfall and temperature changes in the experimental area during the growth stage of the summer maize were detailed in Figure 1.

**Table 1:** Physical properties for experiment soils.

Soil depth (cm)	Mass percentage of particle diameter (%)			Bulk density (g cm^−3^)	pH	Organic matter (g kg^−1^)	Alkali-hydrolyzable nitrogen (mg kg^−1^)	Available phosphorus (mg kg^−1^)	Available potassium (mg kg^−1^)
	Sand	Silt	Clay						
0 ~ 20	15.63	72.31	12.06	1.51	8.02	19.05	19.46	18.99	277.5
20 ~ 40	12.12	76.54	11.34	1.52	7.90	13.85	13.58	14.01	243.5
40 ~ 60	22.13	64.83	13.04	1.47	7.93	7.58	12.52	11.97	182.5
60 ~ 80	14.32	71.62	14.06	1.46	8.07	5.95	10.47	9.86	198.5
80 ~ 100	15.83	70.85	13.32	1.47	8.12	3.41	10.98	10.86	231.5

### Experimental design

**Figure 2:**
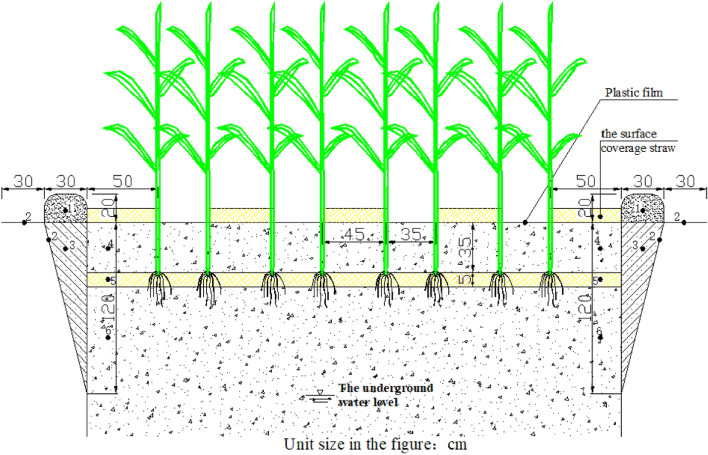
Schematic diagram of straw returning field method. (1) represents soil fencing to prevent water overflow. (2) represents polyethylene film to prevent the interaction of water and fertilizer. (3) represents backfill soil. (4) represents soil tillage layer. (5) represents straw inter-layer with 5 cm. (6) represents heart soil layer.

In this study, the control factors included straw returning method and N application level (pure N content, converted into urea amounts during the application process). Straw returning methods include the surface coverage straw (Treatment B, in which the farmland was turned over to 35 cm, and in second year leveling the farmland, mechanical shallow rake, roller grinding, then use machinery for film-coated planting, finally surfaces were covered with 5 cm thick straw in rows) and the deeply buried straw (Treatment S, in which the farmland was turned over to 35 cm, and 5 cm thick straw layers were manually applied following the autumn harvest. Then, the farmland was raked shallowly and compacted during the second year) (Fig. 2 is a schematic diagram of straw covering or buried deep). The factors of the N applications were set as four specific treatments, which included no N application (Treatment N_0_, 0 kg hm^−2^), a low N application rate of 135 kg hm^−2^ (Treatment N_L_), a medium N application rate of 180 kg hm^−2^ (Treatment N_M_), and a high nitrogen application rate of 225 kg hm^−2^ (Treatment N_H_, local nitrogen application rate). The traditional cultivation was used as a control treatment (Treatment CK, after the autumn harvest in the previous year, the mechanical tilling was about 35 cm, and a large amount of water was used for autumn-irrigation. In early May of the second year, we used mechanical level off the farmland, shallow rake the fields, and roller grinding the fields, then use machinery for film-coated planting. No other field operations were performed, no straw was used, and the N application amount was 225 kg hm^−2^).

There were 9 treatments, each of which was repeated 3 times and randomly arranged. Each plot contained a 3 m protective belt and measured 72 m^2^. The surrounding plots were separated by polyethylene plastic film at buried depth of 1.2 m, with 30 cm of the film left at the top to prevent the water and fertilizer from channeling. The previous crop was summer maize in the experimental field. The straw used in the experiment came from crushed straw after the previous summer's corn harvest. The weight of straw mulching was 1.5 kg m^−2^ and the thickness was 5 cm. The test plots adopted the management modes of the local farmers.The experimental treatment is shown in Table 2.

**Table 2:** Treatments of the experiment.

Treatments	Straw mulching mode	N application amount/(kg hm^−2^)	The amount of P and K /(kg hm^−2^)	Irrigation quota
CK	Traditional cultivation mode	225 (N_H_)	The P fertilizer was calcium superphosphate, which application amount was 150 kg hm^−2^ at the local level (measured by P_2_O_5_). K fertilizer was potassium chloride, and the amount was 45 kg hm^−2^ (measured in K_2_O) at the local level. P-fertilizer, K-fertilizer and 50% N were applied at one time as base fertilizer, and the remaining N was applied at the rapid growth stage	Irrigated by Yellow River water, and the salinity 0.608 g L^−1^. Irrigation was done 3 times in the whole growth period of summer maize. The irrigation quota for a single irrigation was 135 mm. A gasoline pump was used to quantitatively extract water from the canal
BN_0_	Treatment B: Surfaces were covered with 5 cm thick straw in rows after film-coated planting	0 (N_0_)		
BN_L_		135 (N_L_)		
BN_M_		180 (N_M_)		
BN_H_		225 (N_H_)		
SN_0_	Treatment S: Straw layers with 5 cm thicknesses were buried at 35 cm below the ground	0 (N_0_)		
SN_L_		135 (N_L_)		
SN_M_		180 (N_M_)		
SN_H_		225 (N_H_)		

According to the local spraying habit, each community was sprayed evenly within 3 ~ 5 days after the first irrigation of summer maize. The herbicide was 38% AT suspension produced by Dalian Songliao Chemical Company. The amount of herbicide used was local standard, with an average of 2.25 kg hm^−2^ (measured by pure AT quantity, it was the net mass of herbicide). Spray solution evenly with a small hand-held sprayer. Protect yourself when spraying. And don’t spray in the windy weather.

### Plant materials

The summer maize used in this study’s experiment was Junkai 918, which was locally grown varieties and sown using a mechanical seeding process at early May and harvested in late September. The spacing of the summer maize plants was 0.35 m apart, and the row spacing was set as 0.45 m.

### Sampling materials

#### Determination of residual atrazine in soil

According to *Agricultural Chemical Residue Test Criteria* (NY/T 788-2004), the AT content of 0 ~ 5 cm soil samples in each plot was taken as the initial settlement concentration of each treatment one hour after spraying. On the 1st day, 3rd day, 7th day, 21 day, 30 day, 45 day, 90 day and 120 day after spraying, soil samples with a depth of 0 ~ 40 cm were randomly collected. At least 10 points were collected in each treatment. The mass of the soil sample should not be less than 2 kg. Before autumn irrigation, soil samples with a depth of 0 ~ 40 cm should be randomly collected for each treatment. The AT content was determined as the final residual amount of each treatment plot. It was found that AT content in the treated soil with different treatments was low before autumn irrigation. In the second year after autumn irrigation, no AT was detected in the soil or the detected AT content was lower than the detection limit. Therefore, the initial settlement concentration during the 2-year experiment was determined by the above method.

High performance liquid chromatography (Altus A10, PerkinElmer, USA) was used to measure the content of AT in soil. High performance liquid chromatography conditions: chromatographic column was C18 (5 μm, 250 mm length, 4.6 mm diameter). The mobile phase was methanol/water = 70/30 (volume ratio). The flow rate was 1.0 mL min^−1^. The normal column temperature was 40 °C. Detection wavelength of Uv detector was 225 nm, and automatically enters the sample with volume of 10 µL. Under this chromatographic condition, Atrazine retention time was 4.9 min, recovery of standard samples ranged from 92.6 to 106.5%, and the detection volume was 0.02 mg kg^−1^. To draw the standard curve of the standard sample, the x-coordinate is AT standard solution injection concentration (mg L^−1^) and the y-coordinate is the peak area. The linear regression equation is: *y* = 120 071*x* − 251.14, *R*^2^ = 0.999.

#### Testing steps

5.00 (± 0.001) g samples were taken. 50 mL extract was added into a 100 mL centrifuge tube for ultrasonic extraction for 30 min. The supernatant was centrifuged at 4000 r min^−1^ for 5 min. Repeat the step, extract 3 times, combine the supernatant, and rotate evaporation to 35 mL at 35 °C. The organic phase was extracted 3 times with a mixture of dichloromethane and petroleum ether (volume ratio was 35:65), and filtered into a rotating evaporation flask with anhydrous sodium sulfate. Rotate to dry at 35 °C. Methanol was added to 2.0 mL and filtered through a 0.45 μm filter. High performance liquid chromatography was used for determination.

#### Determination of soil organic matter content

In the mature period of summer maize, soil drill was used to take samples in each plot according to the S-type 5 points soil extraction method. Measure soil organic matter content. The 0 ~ 1 m test soil was divided into 5 layers, which was 0 ~ 20 cm, 20 ~ 40 cm, 40 ~ 60 cm, 60 ~ 80 cm and 80 ~ 100 cm, respectively. Take out the same layer of soil sample and mix well. According to Agricultural Chemical Residue Test Criteria of *Forest soil organic matter determination and c/N ratio calculation* (LY/T 1237–1999), Potassium dichromate oxidation-external heating method was used to measure soil organic matter content.

### Statistical design

All data obtained within the individual years were processed using Excel 2010, presented as mean ± standard deviation (SD, n = 3), and analyzed by IBM SPSS Statistics 20. A one-way ANOVA was applied to check the significance of the treatments during the study period (*P* < 0.05), followed by Tukey HSD's multiple range tests. Excel 2010 was used to draw the graph.

## Results and analysis

### Effects of nitrogen application combined with straw returning on the digestion of AT in soil

The results of AT digestion in soil of each treatment were shown in Table 3 (the changing trend of AT digestion in 2 years was basically the same with the time after spraying, and only the experimental data of 2018 were listed here). From the analysis of nitrogen application level, compared with no nitrogen application (Treatment N_0_), nitrogen application significantly promoted AT degradation (*P* < 0.05). In the case of straw cover treatment, the AT digestion rate increased with the increase in nitrogen application rate, and the highest was the conventional nitrogen application level (BN_H_). Under the deeply buried straw treatment, the AT digestion rate increased first and then decreased with the increase of nitrogen application rate, and the highest was the medium nitrogen level (SN_M_). Under the same nitrogen application level, the AT digestion rate of deeply buried straw was 0.2 to 6.2 percentage points higher than that of surface coverage straw. From the analysis of straw mulching methods, different straw mulching methods had different effects on soil AT digestion rate. The AT digestion effect of straw buried deeply was better, which could improve the AT digestion rate of soil to a certain extent. At the mature stage of summer maize (about 100 days after spraying), the AT digestion rate of different treatments was different. SN_M_ treatment had the highest AT digestion rate, averaging 99.89% in 2 years, which was 3.6% higher than CK treatment. BN_0_ treatment had the lowest AT digestion, with an average of 91.76% in 2 years, which was 5.1% lower than CK treatment.

**Table 3:** Dynamic degradation change of AT in soil.

Years	Treatments	Days after spraying/d	0	1	7	14	30	45	90	100
2018	CK	Residues (mg kg^−1^)	4.096	3.197	1.714	1.337	0.851	0.528	0.324	0.152
		Digestion rate (%)		21.95	58.15	67.36	79.22	87.11	92.09	96.29
	BN_0_	Residues (mg kg^−1^)	4.113	3.338	1.855	1.465	1.132	0.852	0.592	0.339
		Digestion rate (%)		18.84	54.90	64.38	72.48	79.29	85.61	91.76
	BN_L_	Residues (mg kg^−1^)	4.022	3.191	1.777	1.288	1.015	0.707	0.429	0.196
		Digestion rate (%)		20.66	55.82	67.98	74.76	82.42	89.33	95.13
	BN_M_	Residues (mg kg^−1^)	3.995	3.102	1.652	1.135	0.945	0.639	0.316	0.112
		Digestion rate (%)		22.35	58.65	71.59	76.35	84.01	92.09	97.20
	BN_H_	Residues (mg kg^−1^)	3.921	2.982	1.611	1.077	0.822	0.506	0.221	0.097
		Digestion rate (%)		23.95	58.91	72.53	79.04	87.10	94.36	97.52
	SN_0_	Residues (mg kg^−1^)	4.142	3.301	1.809	1.292	1.094	0.721	0.416	0.264
		Digestion rate (%)		20.30	56.33	68.81	73.59	82.59	89.96	93.63
	SN_L_	Residues (mg kg^−1^)	4.023	3.098	1.762	1.195	0.972	0.657	0.328	0.151
		Digestion rate (%)		22.99	56.20	70.30	75.84	83.67	91.85	96.25
	SN_M_	Residues (mg kg^−1^)	4.065	2.993	1.587	1.092	0.809	0.398	0.139	0.012
		Digestion rate (%)		26.37	60.96	73.14	80.10	90.21	96.58	99.70
	SN_H_	Residues (mg kg^−1^)	3.914	2.971	1.601	1.068	0.812	0.501	0.202	0.096
		Digestion rate (%)		24.09	59.10	72.71	79.25	87.20	94.84	97.54

To further study the coupling effect of straw mulching and nitrogen application on AT digestion, the paper analyzed the change of AT digestion half-life. The regression results of AT residue in each treatment and spraying time were shown in Table 4. The AT dynamic digestion of the treated soils accords with the first order dynamic equation. The determination coefficients *R*^2^ of the fitting equations were all greater than 0.925, to a significant level (*P* < 0.05). The fitting equation could describe the relationship between soil AT residue and time well. In addition to CK treatment, the half-life of AT digestion in 2019 was shortened by 0.8 to 2.6 days compared with that in 2018. SN_M_ treatment had the shortest AT digestion half-life, with an average of 15.4 day in 2 years, 3.9 day shorter than CK treatment. BN_0_ treatment had the longest AT digestion half-life, with an average of 25.6 day in 2 years, which was 6.3 day more than CK treatment. In straw coverage treatment, the half-life of AT digestion decreased with the increase of nitrogen application rate, and BN_H_ was the shortest. In the straw deeply buried treatments, the half-life of AT digestion tended to be shorter first and longer later with the increase of nitrogen application level, and SN_M_ was the shortest in 2 years. At the same N application level, the half-life of AT digestion under straw deeply buried in 2 years was 1 ~ 7.2 day shorter than that under straw surface coverage. However, there were no significant differences in the half-life of AT digestion among SN_H,_ BN_H_ and CK treatments (*P* > 0.05).

**Table 4:** Kinetic equation of soil AT dissipation under different straw mulching methods and nitrogen application rate.

Years	Treatments	Digestion kinetic equation	Determinate coefficient *R*^2^	Half-life T_0.5_/day	Years	Digestion kinetic equation	Determinate coefficient *R*^2^	Half-life T_0.5_/day
2018	CK	*y* = 2.783*e*^*−*0.035 2*x*^	0.977	19.7b	2019	*y* = 2.763e^−0.036 7*x*^	0.981	18.9b
	BN_0_	*y* = 2.688e^−0.026 0*x*^	0.935	26.7e		*y* = 2.684e^−0.028 3*x*^	0.953	24.5d
	BN_L_	*y* = 2.632e^−0.028 3*x*^	0.937	24.5de		*y* = 2.642e^−0.029 9*x*^	0.951	23.3cd
	BN_M_	*y* = 2.536e^−0.031 2x^	0.947	22.1c		*y* = 2.525e^−0.032 6*x*^	0.952	21.3c
	BN_H_	*y* = 2.576e^−0.036 7*x*^	0.961	18.9b		*y* = 2.607e^−0.040 4*x*^	0.960	17.1b
	SN_0_	*y* = 2.641e^−0.027 4*x*^	0.934	25.3de		*y* = 2.645e^−0.029 5*x*^	0.953	23.5cd
	SN_L_	*y* = 2.587e^−0.030 1*x*^	0.949	23.1cd		*y* = 2.564e^−0.032 6*x*^	0.963	21.3c
	SN_M_	*y* = 2.747e^−0.041 4*x*^	0.966	16.7a		*y* = 2.920e^−0.049*x*^	0.925	14.1a
	SN_H_	*y* = 2.677e^−0.036 7*x*^	0.970	18.9b		*y* = 2.712e ^-0.039 5*x*^	0.977	17.5b

*y* is the residual amount of AT in the soil, mg kg^−1^; *x* is the time after spraying, day.

The final residual amount of AT in soil has important practical significance for its safe use and risk assessment of farmland ecological environment. The final soil AT residues of each treatment in the study period were shown in Table 5. It could be seen from Table 5, before autumn irrigation in 2018, only BN_H_, SN_M_ and SN_H_ treatments had final residues of AT lower than 0.02 mg kg^−1^ or had been completely digested. There were unequal amounts of AT residues in other treatments. Before autumn irrigation in 2019, AT residues in soil of treatment BN_M_, BN_H_, SN_M_ and SN_H_ were lower than 0.02 mg kg^−1^ or completely digested. Other treatments still had AT residue. There were much AT residues of treatment CK in 2 years. Except for treatment CK, AT residues of different straw returning and nitrogen application treatments in 2019 were all lower than those in 2018, with a drop of more than 5.4%. This may be related to the improvement of soil physical and chemical properties by the positioning of straw returning in 3 consecutive years (2017–2019), which needs further study. Straw returning combined with nitrogen application reduced AT residues in soil to a certain extent and reduced the risk of AT pollution in farmland environment. SN_M_ treatment effect was better.

**Table 5:** The final residue of AT in soil for different treatments.

Residues/mg kg^−1^	CK	BN_0_	BN_L_	BN_M_	BN_H_	SN_0_	SN_L_	SN_M_	SN_H_
In 2018	0.052	0.132	0.072	0.047	< 0.02	0.099	0.056	< 0.02	< 0.02
In 2019	0.066	0.125	0.068	< 0.02	< 0.02	0.078	0.047	< 0.02	< 0.02

The detection limit of AT in this study was 0.02 mg kg^−1^.

### Effects of straw returning combined with nitrogen application on SOM content

**Figure 3:**
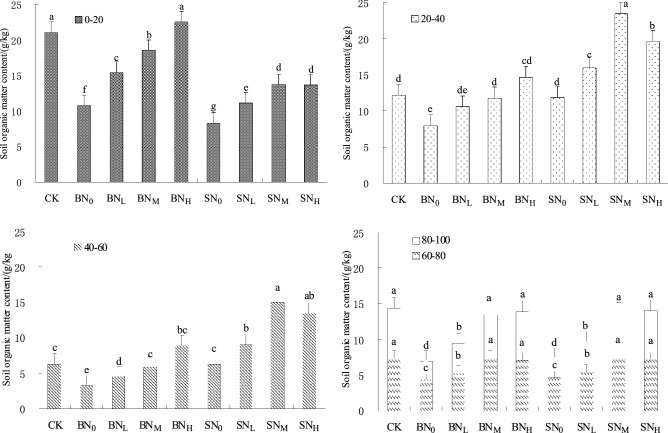
Effects of different treatment on average content of SOM in soil layers.

The spatial distribution trend of SOM content in 2 years was basically the same. The mean value of SOM content of each soil layer in 2 years was taken for analysis. Figure 3 showed the spatial distribution of SOM content in different soil layers under different treatments during summer maize maturity. Compared with no nitrogen application, nitrogen application significantly increased SOM content of each soil layer (*P* < 0.05). The SOM content of surface coverage straw and CK treatments decreased with the deepening of soil depth. The trends were basically similar. It was mainly distributed in 0 ~ 40 cm soil layer, accounting for 68.5% ~ 82.4% of the total SOM content of 1 m soil. With the deepening of soil depth, the SOM content of straw deeply buried treatments increased first and then decreased, and the SOM content of 0 ~ 20 cm soil layer was lower than that of the corresponding surface coverage straw treatments. In the deeply buried straw treatments, the main soil layer of SOM was moved down to the soil layer of 20 ~ 60 cm, and its SOM content accounted for 71.2% ~ 87.3% of the total soil content of 1 m soil. Under the condition of surface coverage straw, SOM content of 4 nitrogen application treatments increased with the increase of nitrogen application rate, and the maximum content was high nitrogen level treatment (BN_H_). However, compared with the straw surface covering treatments, the changing trend of SOM content in straw deep buried treatments with nitrogen application level was significantly different: the SOM content in soil layers of 20 ~ 4 0 cm and 40 ~ 60 cm increased firstly and then decreased with the increase of nitrogen application rate, and SN_M_ treatment was the largest. SOM content in 0 ~ 20 cm soil layer increased with the increase of nitrogen application rate, and SN_H_ treatment was the largest. Straw buried deeply accumulated in the soil layer (20 ~ 40 cm and 40 ~ 60 cm) near the straw inter-layer, and SN_M_ treatment had the highest content, which was 82.6% and 67.7% higher than CK treatment, and was 60.4% and 69.4% higher than BN_H_ treatment, respectively. SOM content of surface coverage straw treatments was accumulated in 0 ~ 20 cm soil layer, and BN_H_ treatment had the highest, which increased by 14.2% and 24.1% on average compared with CK and SN_H_ treatment. There was no significant difference in SOM content of soil layer over 60 cm under different treatments (*P* > 0.05).

### Relationship between half-life of AT digestion and SOM content

The digestion of AT in soil depends not only on its own properties but also on soil nutrients. Under different tillage modes of straw returning and nitrogen application, a single regression analysis was conducted on the half-life of AT digestion and SOM content in 0 ~ 40 cm soil layer, and the results were shown in Table 6.

**Table 6:** Correlation between half-life of atrazine and soil organic matter content.

Years	Soil depth/cm	Fitting equation	Determinate coefficient *R*^2^
In 2018	0 ~ 20	T_0.5_ = 35.40 − 0.981SOM	0.260
	20 ~ 40	T_0.5_ = 33.09 − 0.895SOM	0.914
In 2019	0 ~ 20	T_0.5_ = 25.06 − 0.328SOM	0.396
	20 ~ 40	T_0.5_ = 29.05 − 0.623SOM	0.968

As could be seen from Table 6, the half-life of AT digestion showed different degrees of negative correlation with SOM content in different soil layers, and the correlation with SOM content in 20 ~ 40 cm soil layer was better. The determinate coefficient *R*^2^ of 2-year fitting relationship were 0.914 and 0.968, respectively. The correlation between SOM content and half-life of AT digestion was poor in 0 ~ 20 cm soil layer. In terms of year to year, the correlation coefficient between SOM content and half-life of AT digestion in 2019 was higher than that in 2018. It indicated that SOM content in 20 ~ 40 cm soil layer has a greater impact on the half-life of AT digestion than that in 0 ~ 20 cm soil layer.

To further reveal the effects of SOM content in different soil layers on the half-life of AT digestion, SPSS 20.0 was used for stepwise regression analysis of the half-life of AT digestion and SOM content in the above 2 soil layers (set the threshold of introducing variables at *α* = 0.05 and removing variables at *β* = 0.10). The *F*-test values of stepwise regression variance between the half-life of AT digestion and SOM content in different soil layers reached extremely significant level (*α* = 0.01) in 2 years. The partial regression coefficients were (− 0.446, − 0.867) and (− 0.331, − 0.895) in 2 years, respectively. Therefore, SOM content in 20 ~ 40 cm soil layer had a greater impact on the half-life of AT digestion than SOM content in 0 ~ 20 cm soil layer. The partial regression coefficient of SOM content in 20 ~ 40 cm soil layer reached a significant level of 0.05. It was negatively correlated with the half-life of AT digestion, which was consistent with the results of single regression analysis. Single regression and stepwise regression analysis between the half-life of AT digestion and SOM content in different layers showed that under different straw returning methods, AT digestion was significantly affected by SOM content in 20 ~ 40 cm layer.

## Discussion

Compared with no nitrogen application (treatment N_0_), nitrogen application significantly promoted the degradation of AT. And the effect of straw buried deeply was better than that of surface coverage straw treatment under the same nitrogen application level. The degradation rate of SNM was the fastest, which increased by 5.3% on average compared with CK treatment in 2 years. The variation of soil AT residue with time was in accordance with the first-order kinetic equation, and the determination coefficients *R*^2^ were all greater than 0.925 (*P* < 0.05). The equation could describe the change of soil AT digestion well, which was basically consistent with the results of previous studies^10,12^. The 2-year half-life of AT digestion was different in different treatments, and SN_M_ treatment was the shortest, which was 3.9 d shorter than CK treatment. However, there was no significant difference in the half-life of AT digestion between high nitrogen treatments (BN_H_ and SN_H_) and CK treatments (*P* > 0.05).

Straw returning had been widely used as a tillage measure to improve soil environment^21^, and combined with nitrogen application could significantly increase SOM content^22^. However, this study found that the SOM content of different soil layers under different treatments was different to some extent, and not all soil layers had increased SOM content. Straw mulching was beneficial to SOM surface polymerization and increased with the increase of nitrogen application rate. The SOM content in the surface layer of summer maize at the maturity stage decreased compared with the initial soil content, but it was still significantly increased compared with treatment CK (*P* < 0.05). This was because that treatment CK had no exogenous carbon and nitrogen supply in the middle and late growth of summer maize, and the growth of summer maize would further consume the original SOM in the soil. The content of SOM in CK treatment decreased gradually. Surface coverage straw treatments and nitrogen application reduced soil C/N to a certain extent and promoted straw decomposition. It could not only provide carbon and nitrogen for the growth of summer maize, but also provide carbon sources and nutrients for the growth of microorganism, so as to realize soil nutrient recycling and benefit the accumulation of SOM content.

Compared with the surface coverage straw and CK treatments, straw buried deeply significantly increased the SOM content of soil layer (20 ~ 40 cm and 40 ~ 60 cm) near the straw inter-layer (*P* < 0.05), but SOM content in 0 ~ 20 cm soil layer decreased. Under the condition of straw deeply buried, the SOM content of 0 ~ 20 cm soil layer was gradually consumed by crops and microorganisms with no exogenous replenishment, resulting in a gradual decrease of SOM content in the surface soil layer. The straw crushed in the soil layer near the straw barrier could fully contact with the lower soil, improve the soil aeration, and provide sufficient carbon source for the soil. Application of appropriate nitrogen fertilizer could cause a strong positive excitation effect^23^, promote straw decomposition in the soil, supply carbon and nitrogen sources for the soil, and improve soil enzyme activity^24^, which was conducive to SOM accumulation, and SN_M_ treatment effect was better. Under the same nitrogen application level, there was no significant difference in SOM content of soil layers more 60 cm treated by different straw mulching methods (*P* > 0.05), which was similar to the results obtained by Xie Jun et al^25^. It was because that the soil layer was deeper than 60 cm, soil aeration, microbial number and activity, and nutrients were poor, resulting in the SOM of the upper layer was difficult to transport into and accumulate in the deep soil.

In this study, single regression and stepwise regression analysis were conducted through SOM content of different soil layers and the half-life of AT digestion. Regression results showed that the half-life of AT digestion had a good correlation with SOM content in 20 ~ 40 cm soil layer (*R*^2^ > 0.956). The partial correlation coefficients of 2-year stepwise regression were all greater than 0.867, and SOM content in 20 ~ 40 cm soil layer had a great influence on AT digestion. The reason might be that with the passage of the summer maize growth period, SOM content and other nutrients on the surface were gradually consumed by summer maize, and there was no exogenous replenishment. Combined with the comprehensive effects of surface irrigation, rainfall and other factors, the residual AT gradually leached below the topsoil layer. SOM content in 20 ~ 40 cm soil layer was high, which determined the digestion rate of AT and affected the digestion half-life. It was because that straw returning combined with nitrogen fertilizer changed soil C/N, increased SOM content, promoted the formation of soil aggregates, and increased the stability of soil straw^26^, which was conducive to the adsorption and digestion of AT. SOM could effectively alleviate the toxic effect of AT on soil microorganisms^12^, improve the number and activity of soil microorganisms, enhance microbial metabolism and accelerate the degradation of AT^27^, thus shortening the half-life of AT digestion. In addition, our team found in previous studies that the straw deeply buried tillage mode could effectively improve the root environment of summer maize and contribute to the formation of developed roots^28^. This was beneficial to promote the degradation of AT and shorten the half-life of AT digestion. This might be because plants with developed roots could promote the adsorption and degradation of organic pollutants by rhizosphere microbial flora^29^. Organic pollutants were converted into small molecules such as sugars and amino acids that could be directly absorbed by plant roots to improve the degradation rate of organic pollutants by microorganisms^30^. In this study, it was analyzed the effects of straw mulching combined with nitrogen application on SOM content and AT digestion, and initially revealed the relationship between SOM content and AT digestion half-life. However, the specific mechanism of SOM content and AT digestion in different soil layers needed to be further studied.

## Conclusion

Compared with conventional fertilization tillage, surface coverage straw combined with nitrogen significantly increased SOM content in 0 ~ 20 cm soil layer (*P* < 0.05). while SOM content in the soil layer near the inter-layer was significantly increased by nitrogen application combined with straw buried deeply (*P* < 0.05), and effectively improved SOM distribution in plough layer.

Under straw returning and nitrogen application mode, the change of AT residue over time was in accordance with the first-order kinetic equation. Among all the treatments, SN_M_ treatment had the fastest digestion and the shortest half-life digestion. The average digestion rate of SN_M_ was 5.3% higher than CK treatment, and the average half-life of SN_M_ was 3.9 d shorter than CK.

Under straw mulching, the half-life of AT digestion was significantly correlated with SOM content in 20 ~ 40 cm soil layer. AT digestion was effectively promoted by straw deeply buried combined with medium nitrogen treatment (SN_M_). Therefore, straw deeply buried and medium nitrogen application was used as the tillage and nitrogen application mode to improve AT digestion rate and farmland environment in Hetao Irrigation District.

## Data Availability

All data generated or analysed during this study were included in the article. (We confirmed the data used to support the findings of this study were available from the corresponding author upon request).
